# P-1614. Verigene's Impact on Time to Preferred Antibiotics in Gram Positive Bacteremia

**DOI:** 10.1093/ofid/ofae631.1781

**Published:** 2025-01-29

**Authors:** Meaghan Martinez-Palmer, Alison Robins

**Affiliations:** Baylor College of Medicine, Houston, Texas; Baylor College of Medicine, Michael E. DeBakey VA Medical Center, Housotn, Texas

## Abstract

**Background:**

Utilization of rapid identification methods for positive blood cultures has been instrumental in the efforts of antimicrobial stewardship programs (ASPs). Studies have shown that the use of molecular testing techniques on positive blood cultures, which identify select bacteria and their genetic resistance, have enabled timelier initiation of targeted therapy.

Type of Bacteremia

**Figure:**
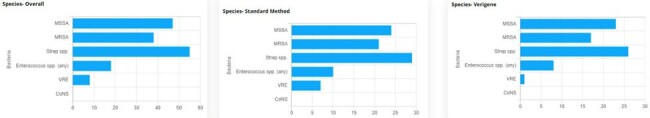

Species of bacteremia identified in both the standard identification period and the identification by Verigene period

**Methods:**

Data was collected via chart review over two separate 6-month periods (7/1/2021- 1/1/2022 and 7/1/2022-1/1/2023), with Verigene BCID having been implemented in June 2022. The primary endpoint was the time to preferred therapy for organisms reported in the Verigene gram-positive panel, including MSSA, MRSA, Enterococcus species (faecalis and faecium), VRE, and Streptococcus species. Demographic variables, the source of bacteremia when established (i.e., skin/soft tissue, urinary, etc), as well as interventions for source control were also collected.

Source of Bacteremia

**Figure:**
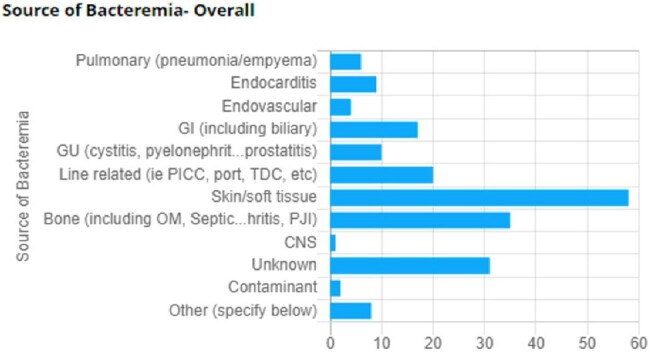

**Results:**

In this study, we analyzed a total of 168 cases of gram-positive bacteremia. Among these, 94 cases occurred between 7/1/2021-1/1/2022 with identification via the standard method, while 74 cases occurred between 7/1/2022-1/1/2023 with identification via Verigene BCID. The average age of patients at the onset of bacteremia was 68 years, with a predominance of males (94.6%). Common underlying comorbidities included diabetes (50%) and coronary artery disease (48.7%).

Regarding bacteremia etiology, 29.8% were caused by MSSA, 22.6% by MRSA, 32.7% by Streptococcus species, 11.3% by Enterococcus spp, and 6% by VRE. The most common primary source of bacteremia was identified as skin and soft tissue (34.5%).

The time to administration of preferred antibiotics, as defined by our ASP recommendations, showed improvement across all gram-positive bacteremia types, decreasing from a mean of 61.5 hours with standard identification methods to 36 hours with Verigene identification. Time to preferred therapy decreased from 81.4 hours to 39.8 hours for MSSA bacteremia, and from 61.9 hours to 39.5 hours for Strep bacteremia.

Time to Preferred Antibiotics

**Figure:**
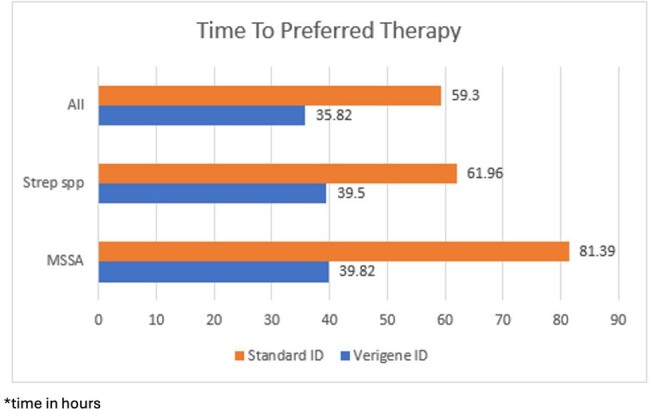

Time in hours to preferred antibiotics for all cases analyzed, Streptococcus species, and MSSA.

**Conclusion:**

The implementation of rapid blood culture identification utilizing Verigene BCID technology lead to a significant improvement in time to preferred antibiotics in cases of gram positive bacteremia.

**Disclosures:**

**All Authors**: No reported disclosures

